# Stimulus familiarity modulates functional connectivity of the perirhinal cortex and anterior hippocampus during visual discrimination of faces and objects

**DOI:** 10.3389/fnhum.2014.00117

**Published:** 2014-03-04

**Authors:** Victoria C. McLelland, David Chan, Susanne Ferber, Morgan D. Barense

**Affiliations:** ^1^Department of Psychology, University of TorontoToronto, ON, Canada; ^2^Rotman Research InstituteBaycrest, Toronto, ON, Canada

**Keywords:** functional connectivity, perirhinal, hippocampus, perception, semantic memory, familiarity

## Abstract

Recent research suggests that the medial temporal lobe (MTL) is involved in perception as well as in declarative memory. Amnesic patients with focal MTL lesions and semantic dementia patients showed perceptual deficits when discriminating faces and objects. Interestingly, these two patient groups showed different profiles of impairment for familiar and unfamiliar stimuli. For MTL amnesics, the use of familiar relative to unfamiliar stimuli improved discrimination performance. By contrast, patients with semantic dementia—a neurodegenerative condition associated with anterolateral temporal lobe damage—showed no such facilitation from familiar stimuli. Given that the two patient groups had highly overlapping patterns of damage to the perirhinal cortex, hippocampus, and temporal pole, the neuroanatomical substrates underlying their performance discrepancy were unclear. Here, we addressed this question with a multivariate reanalysis of the data presented by Barense et al. (2011), using functional connectivity to examine how stimulus familiarity affected the broader networks with which the perirhinal cortex, hippocampus, and temporal poles interact. In this study, healthy participants were scanned while they performed an odd-one-out perceptual task involving familiar and novel faces or objects. Seed-based analyses revealed that functional connectivity of the right perirhinal cortex and right anterior hippocampus was modulated by the degree of stimulus familiarity. For familiar relative to unfamiliar faces and objects, both right perirhinal cortex and right anterior hippocampus showed enhanced functional correlations with anterior/lateral temporal cortex, temporal pole, and medial/lateral parietal cortex. These findings suggest that in order to benefit from stimulus familiarity, it is necessary to engage not only the perirhinal cortex and hippocampus, but also a network of regions known to represent semantic information.

## Introduction

The medial temporal lobe (MTL) is comprised of several highly-interconnected structures including the hippocampus, entorhinal, perirhinal, and parahippocampal cortices. These regions have generally been thought to exclusively support functions related to long-term declarative memory (Squire et al., 2004; Squire and Wixted, 2011). Recently, however, it has become apparent that some of these structures play an important role in other cognitive functions, such as certain perceptual processes. For example, the perirhinal cortex is important for making perceptual discriminations among items that share a large number of overlapping features, particularly when it is necessary to process conjunctions of these features (Bussey and Saksida, 2002; Barense et al., 2005, 2007; Bartko et al., 2007; Devlin and Price, 2007; O'Neil et al., 2009). The involvement of the perirhinal cortex in perception has been demonstrated for a variety of stimulus classes with complex features, including inanimate objects, but also faces (Lee et al., 2005a,b, 2008; Barense et al., 2010a). Based on this evidence for a perceptual function of the perirhinal cortex, as well as on findings that the hippocampus is involved in the discrimination of three-dimensional scenes (Lee et al., 2012), it has been argued that the recruitment of structures within the MTL depends more on the nature of the items being represented, as opposed to whether the task explicitly targets long-term memory (Bussey and Saksida, 2007; Graham et al., 2010).

The perirhinal cortex, located caudally in relation to the temporal pole, but also exhibiting strong connectivity with more posterior inferior temporal visual regions (Suzuki and Amaral, 1994), is well-placed to serve as an interface between perception and semantic memory. Within the domain of semantic memory, the perirhinal cortex seems to be particularly important for storing and binding conceptual information about objects (Murray and Bussey, 1999; Taylor et al., 2006; Chan et al., 2011), and also for differentiating between similar members of a single category that share many conceptual features, particularly among living things (Moss et al., 2005; Tyler et al., 2013). It is one of several regions affected by semantic dementia—a neurodegenerative disease characterized by progressive loss of semantic knowledge and degeneration of the anterior temporal lobes (Hodges et al., 1992)—and perirhinal cortex atrophy in the disorder has been associated with deficits on a large battery of tasks assessing conceptual knowledge (Davies et al., 2004).

There is evidence that semantic memory and perceptual functions interact, such that the process of perceiving everyday items like faces and objects is influenced by stimulus familiarity (in this case, the term “familiarity” refers to the degree to which participants know and have previous experience with the stimuli, and should not be confused with the concept denoting a feeling of knowing without vivid recollection of contextual details; e.g., Yonelinas, 2002). For example, the perirhinal cortex mediates aspects of the interaction between perception and conceptual knowledge, as this structure is necessary for the perception and detection of familiar object feature configurations in figure-ground tasks (Barense et al., 2012; Peterson et al., 2012). In general, familiarity significantly affects the efficiency with which items such as faces and objects are recognized (Bülthoff and Newell, 2006), and familiarity can alter the default level at which classes of objects are categorized, such that participants are able to more quickly categorize familiar items at an individual level (e.g., Bill Clinton, the Eiffel Tower), but are slower to categorize these same items at the basic level (e.g., a human face, a building), which is the reverse of the pattern typically seen with unfamiliar stimuli (Anaki and Bentin, 2009). Perceptual expertise, which is a form of familiarity that results from the acquisition of experience with particular natural classes of objects outside of a laboratory setting, can alter even the neural representation of these object categories, such that they come to be processed more in lateral occipital cortex and fusiform gyrus instead of in earlier visual cortical regions (McGugin et al., 2012; Wong et al., 2012).

Items with which participants have had some previous experience automatically prompt the retrieval of more related semantic or general conceptual information than do novel items. For example, viewing unfamiliar faces will result in retrieval of some limited conceptual information about the individuals' age, sex, and emotional expression, whereas viewing familiar faces is accompanied by retrieval of more detailed identity-specific information (Bruce and Young, 1986). Moreover, faces appear to be a type of stimulus that is particularly associated with semantic information, relative to other personal characteristics such as voices (Barsics and Brédart, 2012). The retrieval of semantic material associated with a stimulus appears to be spontaneous, with semantic information retrieved automatically even when participants are engaged in another irrelevant task (Jung et al., 2013). Semantic processes are therefore likely to be involved in any task that contains elements with which participants are familiar.

The automatic retrieval of semantic knowledge associated with familiar stimuli affects performance on perceptual discrimination tasks, even when the completion of these tasks does not overtly require the use of semantic information. In Barense et al. (2010b), two patient groups with differing profiles of temporal lobe damage completed perceptual discrimination tasks that required choosing the odd-one-out among concurrently-presented complex stimuli. The stimuli were either everyday familiar objects (e.g., cars) or unfamiliar, novel objects (e.g., “greebles,” Gauthier and Tarr, 1997). Results revealed differential effects of stimulus familiarity in amnesic patients with non-progressive MTL damage (“MTL amnesics”) vs. patients with neurodegeneration of the temporal lobes caused by semantic dementia (“SD patients”). Whereas both healthy controls and MTL amnesics benefitted from stimulus familiarity, SD patients did not show this facilitation. The MTL amnesics did in fact perform significantly worse than controls when discriminating among both novel and familiar stimuli, but their deficit for familiar stimuli was attenuated by their relatively unimpaired access to semantic memory. However, as the lesions in both the MTL and SD patients were widespread and variable, and both had significant damage to the perirhinal cortex and temporal pole, it was difficult to draw conclusions about the specific brain regions responsible for these differential effects.

**Figure 1:**
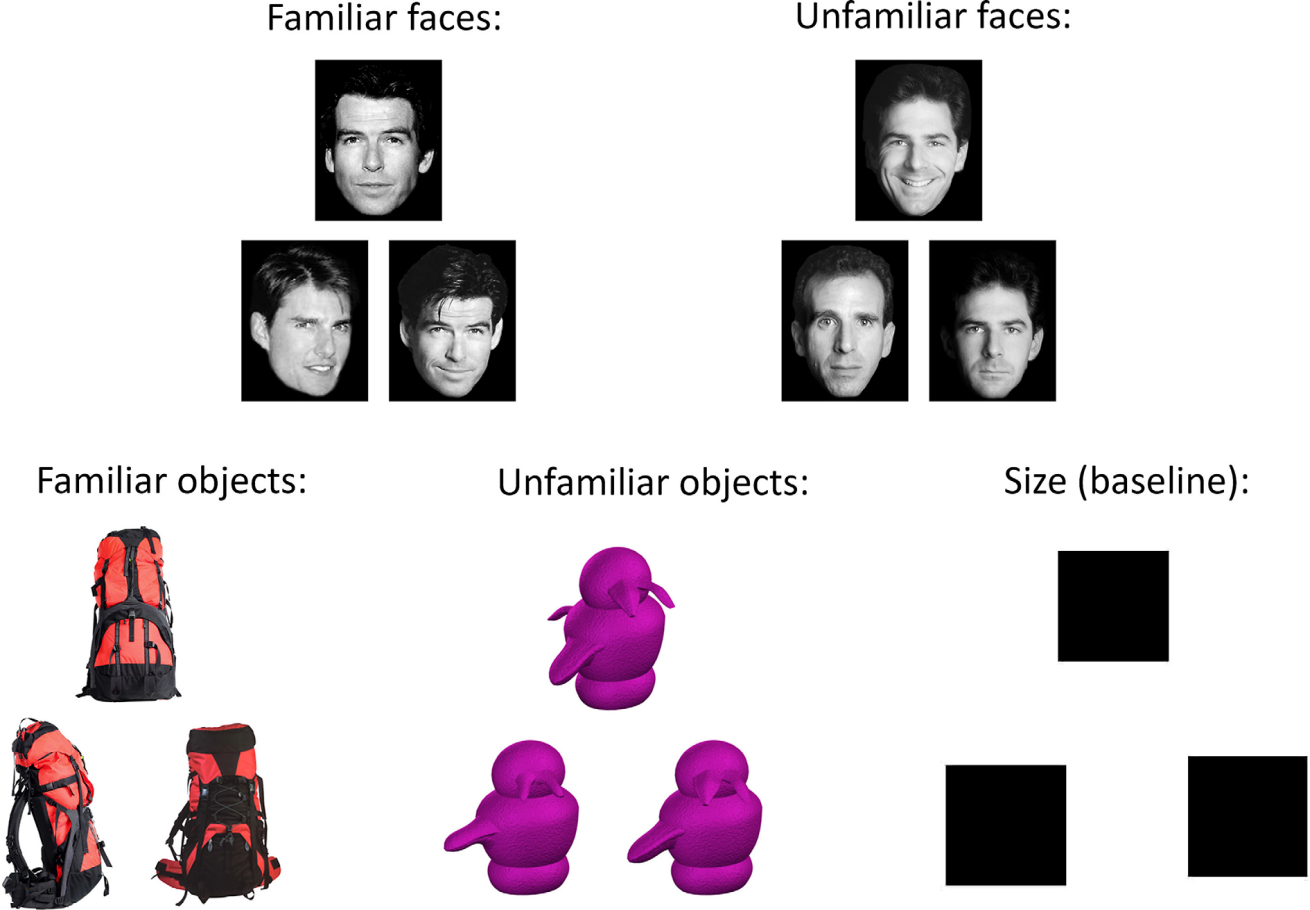
**Example stimuli from five conditions in the oddity discrimination task.** On each trial, participants were presented with a set of three trial-unique items. Two of these items were pictures of the same stimulus shown from different viewpoints, while the remaining item was a picture of a slightly different item shown from another viewpoint. Participants were asked to select the different item (the “odd-one-out”). Stimuli on each trial were either familiar (famous faces or everyday objects) or novel (unfamiliar faces or greebles). On the size baseline task, two of the squares were the same size, and the third square was slightly smaller or larger; participants were instructed to choose the different-sized square. In the current study, our primary contrast of interest was between the familiar and unfamiliar conditions; consequently the size contrast condition was not analyzed.

To further investigate the neural correlates of familiarity effects in perceptual discrimination tasks, Barense et al. (2011) scanned healthy control participants while they identified the odd-one-out among sets of objects and faces that varied in familiarity (see Figure 1) and found that a number of regions throughout the MTL were sensitive to stimulus familiarity. Specifically, the perirhinal cortex, temporal pole, and anterior hippocampus were all more active bilaterally during discriminations of familiar faces relative to unfamiliar faces. Likewise, the perirhinal cortex and anterior/posterior hippocampus were more active bilaterally for familiar objects relative to unfamiliar objects. This observed activity was not simply a reflection of successful encoding, as these effects were still evident when the analysis was restricted to trials in which participants did not later remember the stimuli in a surprise recognition memory test. However, the perirhinal cortex, hippocampus, and temporal poles cannot be the sole regions underlying the familiarity effects observed in patients with temporal lobe damage (i.e., facilitation from familiar stimuli in focal MTL amnesics, but no such facilitation in SD patients), because these MTL amnesics had suffered damage to all of these structures and yet still showed facilitation from the use of familiar stimuli (Barense et al., 2010b).

Given that brain damage in humans is rarely restricted to discrete anatomical areas of theoretical interest, insight into the differences between these patient groups can be gained by examining how stimulus familiarity affects the broader networks of regions with which the perirhinal cortex, hippocampus, and temporal poles interact. The measurement of functional connectivity is one method of acquiring such information. This technique involves the identification of regions throughout the brain in which changes in activation occur at the same time and at a similar magnitude to the changes in activation in specific regions of interest, or *seeds*. Any areas exhibiting such a correlation are broadly thought to be functionally interacting with the seed regions in some way (Rogers et al., 2007; Friston, 2011). Using these techniques, it has already been established that the functional connectivity of the MTL can be affected by changing task demands (Martin et al., 2011; O'Neil et al., 2012).

In the present study, we examined how the functional connectivity of regions in the perirhinal cortex, temporal pole, and anterior and posterior hippocampus identified by Barense et al. (2011) varied during perceptual discrimination, depending on the degree to which participants were familiar with the items to be discriminated. We hypothesized that for discriminations involving familiar stimuli (relative to unfamiliar stimuli), at least some of these four subregions would exhibit significantly greater interaction with structures thought to represent semantic information, including anterior temporal (e.g., Patterson et al., 2007; Binney et al., 2010; Visser et al., 2010), lateral temporal (e.g., Schmolck et al., 2002; Levy et al., 2004), and inferior parietal cortex (Binder and Desai, 2011; Fairhall and Caramazza, 2013). If confirmed, the findings would offer insight into the performance discrepancies reported between SD patients and MTL amnesics during perceptual discrimination of familiar stimuli (Barense et al., 2010b). To this end, we conducted a partial least squares (PLS) analysis of functional connectivity on the data described in Barense et al. (2011).

## Materials and methods

This study involved a re-analysis of the data presented in Barense et al. (2011) using multivariate statistical imaging techniques, and thus, a full description of the methods can be found there. Accordingly, only the aspects of the experimental design that are relevant to the current analyses are presented here.

### Participants

Eighteen young adult participants (12 female, *M* = 27.3 years old, *SD* = 5.5 years) were recruited. All were right-handed and did not suffer from any neurological abnormalities. All participants gave informed written consent, and this research received ethical approval from the Cambridgeshire Local Research Ethics Committee (LREC reference 05/Q0108/127).

### Procedure

An oddity discrimination paradigm was employed, with each participant completing 405 trials (81 trials per condition, 105 trials in each of the first three runs, and 90 trials in the fourth run). Participants were simultaneously presented with pictures of three items: two of these pictures were of the same stimulus and the other was a picture of a different stimulus. Participants were asked to identify the odd one out (see Figure 1), and indicated their responses by pressing one of three specified buttons on a four-button response box held in the right hand. The sets of stimuli always appeared in the same layout, with two items next to each other and a third item above the other two, though the location of the odd-one-out was counterbalanced across trials. All stimuli were trial-unique and therefore not repeated across trials. The stimuli in each trial belonged to one of four possible experimental conditions: *familiar (famous) faces*, *unfamiliar faces*, *familiar objects*, and *unfamiliar objects (greebles)*. In addition, there was a *size control* condition, in which the stimuli were black squares. The sets of stimuli were each displayed for 5.5 s, during which participants indicated the odd stimulus with a corresponding button press as quickly and as accurately as possible. The inter-trial interval was 0.25 s, except that on every 11th trial there was an additional 0.60 s delay, during which the experimental program checked to ensure that it was synced to the appropriate scanner pulse. Each condition was presented across “mini-blocks” of three trials, such that participants were shown three trials in a row belonging to a single condition before moving on to a mini-block of a different condition. The order of these mini-blocks was fixed for each participant and counterbalanced across participants. This ensured that a given trial type did not always follow the same trial type, allowing decoupling of signal across conditions.

In each of the two face conditions (*familiar* and *unfamiliar faces*), the items presented were grayscale photographs of White human faces displayed on a white background. On each trial, two of the three images were of the same face but shown from different viewpoints, while the third image was a different face shown from yet another viewpoint. The *familiar faces* were famous faces that were likely known to participants, while the *unfamiliar faces* were novel and not known to participants. On each of the 81 trials per condition, 40 of the trials involved female faces and 41 trials involved male faces.

In the two object conditions (*familiar* and *unfamiliar objects*), the stimuli were color photographs of objects. On each trial, all stimuli belonged to the same basic category, with two of the stimuli being the same object depicted from different viewpoints, and the third image showing a second, different object from another viewpoint. The two objects for each trial were chosen so as to have as many overlapping features as possible, in order to prevent participants from being able to make their oddity judgments based upon a single distinguishing feature. The *familiar objects* were commonplace and inanimate items selected from a large database (Hemera Photo-Objects Volumes 1–3), while the *unfamiliar objects* were “greebles” (Gauthier and Tarr, 1997), which are well-studied stimuli that, like faces, have a homogeneous spatial configuration, but with which our participants had no prior exposure or expertise.

In the *size control* condition, the stimuli consisted of three black squares presented in slightly jittered positions, with two of the squares being the same size and the third square being slightly smaller or larger (by a range of 9–15 pixels per side) than the other two.

### MRI parameters

MRI images were acquired on a Siemens 3.0 Tesla Tim Trio MRI scanner. Each participant's anatomical scan was collected using a magnetization-prepared rapidly-acquired gradient echo (MP-RAGE) sequence [repetition time (*TR*) = 2250 ms, echo time (*TE*) = 2.99 ms, flip angle = 9°, field of view = 256 × 240 × 160 mm, matrix size = 256 × 240 × 160 mm, spatial resolution = 1 × 1 × 1 mm]. Functional images were acquired using a T2^*^-weighted echo planar imaging (EPI) sequence with two echoes (spin echo and gradient echo, Schwarzbauer et al., 2010) in attempt to avoid the loss of signal that often occurs when collecting images of the inferior temporal lobes and orbitofrontal cortex (slice thickness = 3 mm, gap = 1 mm, matrix size = 64 × 64, in-plane resolution = 3.5 × 3.5 mm, *TR* = 2000 ms). Spin-echo images are generally less prone to susceptibility artifacts, but in this case the spin-echo data did not reveal any effects that were not already evident in the standard gradient-echo sequence. Consequently, only data from the gradient-echo sequence is described here. Sixteen slices were acquired in an interleaved fashion (one spin-echo and one gradient-echo image per slice, resulting in an effective total of 32 slices), following the temporal lobes and parallel to the long axis of the hippocampus. Because half of the slices were devoted to the spin echoes and the time to acquire each volume was effectively doubled, the brain coverage was focused on temporal regions and our coverage of frontal regions was limited. Each participant completed four functional runs, with the first three runs lasting 630 s in duration, and the fourth run lasting 542 s. The first 5 scans of each run were discarded to allow the MRI signal to reach equilibrium.

### Image pre-processing

Functional MRI images were preprocessed using a standard protocol within Statistical Parametric Mapping software (SPM5, www.fil.ion.ucl.ac.uk/spm/software/spm5). All functional images were realigned to the first image of the first run, and un-warped to correct for distortions in the main magnetic field. All participants' anatomical images were normalized to the Montreal Neurological Institute (MNI) template, and the resulting normalization parameters were then applied to all functional images (resampled at 3 × 3 × 3 mm voxels). The normalized images were then spatially smoothed with an 8 mm full-width half-maximum (FWHM) Gaussian kernel.

### Partial least squares analyses

Spatiotemporal partial least squares correlation was used to analyze the functional MRI data (McIntosh et al., 1996, 2004; McIntosh and Lobaugh, 2004; Krishnan et al., 2011). PLS is a highly-sensitive, covariance-based multivariate technique, similar to principal component analysis (PCA) but instead of operating on the total variance, PLS focuses solely on the covariance between the functional data and the task/experimental design, constraining solutions to be those related to the experimental conditions (McIntosh et al., 2004). Using PLS software (http://www.rotman-baycrest.on.ca/index.php?section=84), we first ran an initial mean-centered task PLS analysis, which identifies patterns of activation that optimally distinguish between experimental conditions. This was done in order to generate the voxel signal intensity data for our subsequent seed PLS functional connectivity analysis. Because our planned contrast for this seed PLS analysis was between the familiar vs. unfamiliar conditions, we were particularly interested in whether the mean-centered task PLS analysis would identify regions that discriminated between familiar and unfamiliar faces/objects. Subsequent seed PLS analyses were conducted to reveal networks functionally connected to the MTL regions found to be responsive to stimulus familiarity in Barense et al. (2011).

### Mean-centered task partial least squares analysis

We initially conducted a mean-centered task PLS analysis (McIntosh et al., 1996, 2004; McIntosh and Lobaugh, 2004), from which we planned to extract signal intensity values from the MTL seed regions identified in the previously-published univariate analysis (Barense et al., 2011). Although the seed-based functional connectivity analyses were of primary interest in the current study, the mean-centered task PLS analyses also allowed us to determine whether an alternate, multivariate approach would also implicate the MTLs in the perceptual discrimination of familiar stimuli, as described by Barense et al. (2011). Mean-centered task PLS is an exploratory, data-driven form of PLS in which no *a priori* contrasts or hypotheses are specified, and a series of *latent variables* (LVs) that optimally account for the covariance between functional data and experimental conditions are derived using singular value decomposition (McIntosh et al., 2004). These LVs, which are similar to eigenvectors in PCA, have three components: a *singular value*, which describes the proportion of the covariance between the task and the functional data accounted for by that particular LV, *voxel saliences*, which identify the distributed spatial pattern of brain voxels that is most related to the effect characterized by that LV, and *task saliences*, which illustrate the extent to which each experimental condition is associated with that pattern of voxels. Effects were assessed over a 16 s temporal window from the onset of each trial. As we were primarily interested in the differences between the four experimental conditions (*familiar faces*, *unfamiliar faces*, *familiar objects*, and *unfamiliar objects*), as opposed to differences between the experimental and control conditions, the *size control* condition was excluded from these analyses. The functional MRI data were transformed into a 72 × 282,440 matrix, in which the rows represented each of the four conditions for each of the 18 participants, while the columns contained the signal values for every voxel at each of the eight 2 s time lags contained within the 16 s temporal window. Singular value decomposition was performed on a mean-centered, columnwise averaged form of this matrix, yielding the three components mentioned above.

The statistical significance of each LV was evaluated with 500 permutation tests, calculated with a threshold of *p* < 0.05 (McIntosh et al., 1996; McIntosh and Lobaugh, 2004). Each permutation test involves random reordering and reassignment of every participant's data to the specified experimental conditions, determining the likelihood of each LV's singular value occurring by chance. Additionally, the reliability of the voxel saliences (i.e., the clusters of brain regions identified by each LV) was measured by bootstrap estimation of their standard errors, entailing random sampling of participants with replacement 300 times, determining which voxel responses appear reliably (and therefore are not heavily influenced by which participants are included in the sample). Here, clusters with bootstrap ratios (BSR) of greater than ±3.5 were classified as reliable (A BSR is approximately equivalent to a *z*-score, and in this case corresponds to a *p*-value of roughly 0.001; McIntosh and Lobaugh, 2004). As PLS analyses are conducted in a single analytic step, correction for multiple comparisons is not required.

### Seed partial least squares analyses

Following this preliminary mean-centered task PLS analysis, we then used a “seed” PLS analysis (McIntosh et al., 2004) to examine the functional connectivity of the four regions identified as being sensitive to stimulus familiarity in Barense et al. (2011). This form of PLS can be used to assess functional connectivity by pinpointing regions across the whole brain in which signal is correlated with that of user-specified seed regions of interest, and determining how this connectivity changes across experimental conditions. In this case, the seed regions were the peak MNI coordinates that had been identified in the univariate contrasts of the familiar vs. the unfamiliar conditions in Barense et al. (2011). These regions included the perirhinal cortex, the anterior hippocampus, the posterior hippocampus, and the temporal pole (one seed in each hemisphere). The voxel signal from each of these seeds was extracted from the mean-centered task PLS result using PLS software's multiple voxel extraction tool, centered on the following peak MNI coordinates identified by Barense et al. (2011): left perirhinal cortex (*x*, *y*, *z* = −33, −12, −27), left anterior hippocampus (*x*, *y*, *z* = −21, −9, −18), left posterior hippocampus (*x*, *y*, *z* = −33, −27, −15), left temporal pole (*x*, *y*, *z* = −36, 18, −27), right perirhinal cortex (*x*, *y*, *z* = 36, −9, −30), right anterior hippocampus (*x*, *y*, *z* = 27, −15, −18), right posterior hippocampus (*x*, *y*, *z* = 33, −33, −12), and right temporal pole (*x*, *y*, *z* = 63, 3, −18), averaging signal intensity across the two nearest neighboring voxels. Peak signal intensity values were extracted for each voxel from lag 3 (6 s from trial onset) of the mean-centered task analysis, as this timepoint corresponds to the typical peak of the hemodynamic response function (cf. O'Neil et al., 2012).

These signal intensity values for each participant were entered in matrix form as the behavioral values in a single non-rotated seed PLS analysis. This 72 × 8 matrix contained four rows for each of the 18 participants, and one column for each of the seed regions. Correlations were computed between this matrix and the matrix of functional MRI data containing all voxel signal values as described earlier for the mean-centered task PLS analysis. The resulting correlation maps were stacked and again analyzed with singular value decomposition. The non-rotated version of PLS, instead of being data-driven, constrains the possible solutions to allow for the explicit testing of hypotheses via specification of *a priori* contrasts. Non-rotated PLS analyses yield one LV corresponding to each specified contrast. Considering that we were primarily interested in the effects of stimulus familiarity, within this model we specified a contrast for each seed investigating whether the functional connectivity of that seed differed between the familiar and unfamiliar conditions (irrespective of whether the stimuli were faces or objects). The significance of the LVs and reliability of the voxel saliences were evaluated in the same manner as the mean-centered task analysis, with 500 permutation tests (*p* < 0.05) and 300 bootstrap estimations (BSR = ±3.5). Peak MNI coordinates from reliable clusters identified in both the mean-centered and seed PLS analyses are reported in Tables 2–4, with anatomical labels assigned using the Automated Anatomical Labeling atlas (Tzourio-Mazoyer et al., 2002). In all figures for both the mean-centered and seed PLS analyses, conditions and brain regions with positive saliences are always displayed in red and those with negative saliences are always displayed in blue.

## Results

### Behavioral results

Accuracy and reaction time (RT) for each of the five conditions are shown in Table 1 (reproduced from Barense et al., 2011). A repeated-measures ANOVA indicated that there was no main effect of stimulus familiarity on participants' accuracy in the discrimination task, *F*_(1, 17)_ = 0.90, *p* = 0.357, indicating that differences in functional connectivity between the familiar and unfamiliar conditions did not stem from factors related to accuracy. However, there was a significant main effect of familiarity on RT, *F*_(1, 17)_ = 41.47, *p* < 0.001, due to the fact that the mean RT for the familiar conditions (*M* = 2813.13, *SE* = 96.56) was significantly faster than for the unfamiliar conditions (*M* = 3048.00, *SE* = 80.50). Despite this effect, it is unlikely that the difference in RT underlies any observed differences in functional connectivity between the familiar and unfamiliar conditions. The mean RT discrepancy of 234 ms is far shorter than a single TR; consequently, peak correlations between functionally interacting regions during both the familiar and unfamiliar conditions would still fall within the same time lag in our PLS analyses.

**Table 1:** **Mean accuracy and reaction time (correct trials only) for each condition (reproduced from Barense et al., 2011)**.

	**Familiar faces**	**Unfamiliar faces**	**Familiar objects**	**Unfamiliar objects**	**Size**
Proportion correct	0.87 (0.07)	0.84 (0.07)	0.77 (0.07)	0.77 (0.09)	0.79 (0.10)
RT (ms)	2616 (415)	2796 (349)	3009 (448)	3300 (386)	2110 (366)

RT, reaction time; standard deviation values are shown in parentheses.

### Mean-centered task PLS analysis

**Figure 2:**
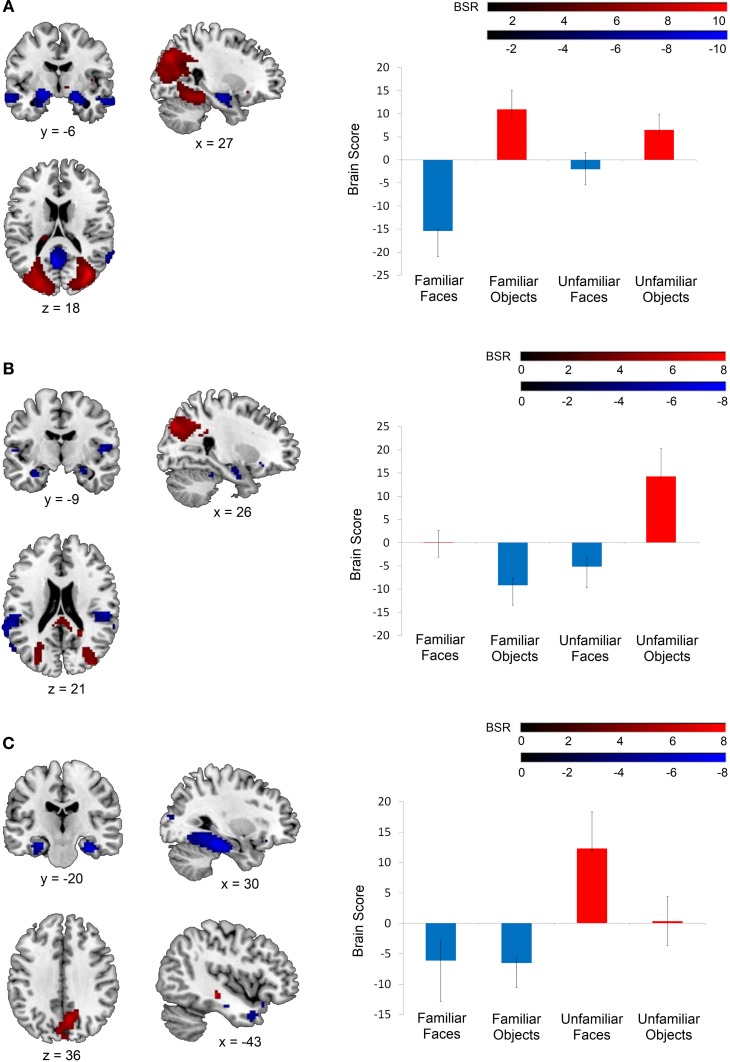
**Latent variables identified in the mean-centered task PLS analysis.** On the left, voxel salience maps are shown. On the right, bar graphs for each of the three LVs illustrate the extent to which each condition corresponds to each of the positively-weighted (shown in red on brain images) and negatively-weighted (shown in blue on brain images) networks. These networks, in the form of voxel salience maps for each LV, are all shown for TR 3, superimposed on the ch2bet template in MRIcron (Rorden et al., 2007). **(A)** LV 1 highlighted the differences between the face and object conditions, **(B)** LV 2 highlighted the difference between the unfamiliar objects condition and the unfamiliar faces and familiar objects conditions, and **(C)** LV 3 identified differences between the familiar and unfamiliar conditions. Maps are thresholded at the equivalent of *p* < 0.05 for visualization purposes. BSR, bootstrap ratio. Error bars reflect 95% confidence intervals.

The mean-centered task PLS analysis yielded three significant LVs (see Table 2). The first LV explained 42.53% of the crossblock covariance (*p* < 0.001), and differentiated between the face and non-face conditions. Two negatively-correlated patterns of activation were identified, one associated with both the *familiar* and *unfamiliar objects* conditions (shown in red in Figure 2A), and another associated with the *familiar faces* condition (shown in blue in Figure 2A). The regions associated more highly with the *familiar faces* condition relative to the *familiar* and *unfamiliar objects* conditions included bilateral clusters in the anterior hippocampus and anterior lateral temporal cortex, which are areas that correspond well to the regions that responded to face familiarity from the univariate general linear model analysis in Barense et al. (2011). The regions associated more with the two object conditions relative to the *familiar faces* condition included large sections of occipital cortex and bilateral fusiform gyrus.

**Table 2:** **Regions associated with the latent variables from the mean-centered task PLS analysis**.

**Latent variable**	Cluster size (*k*)^*^	**Brain region**	***x***	***y***	***z***	**BSR**
*1*	**FAMILIAR OBJECTS AND UNFAMILIAR OBJECTS > FAMILIAR FACES**					
	1170	R Middle occipital gyrus	39	−75	21	10.10
	1001	L Superior occipital gyrus	−21	−81	36	9.37
	463	R Fusiform gyrus	33	−36	−15	8.27
	167	L Fusiform gyrus	−27	−51	−12	4.96
	73	R Inferior frontal gyrus	33	24	−12	4.23
	44	L Thalamus	−18	−27	6	4.22
	20	L Lingual gyrus	−6	−69	−6	3.94
	16	R Insula	42	−12	6	3.91
	17	R Thalamus	12	−21	12	3.86
*1*	**FAMILIAR FACES > FAMILIAR OBJECTS AND UNFAMILIAR OBJECTS**					
	338	Precuneus/Posterior cingulate gyrus	0	−60	21	−7.04
	177	R Hippocampus	21	−9	−15	−6.70
	197	L Hippocampus/Parahippocampal gyrus	−21	−6	−9	−6.52
	311	R Middle temporal gyrus	66	−48	12	−6.39
	101	R Middle temporal gyrus	60	−3	−21	−6.05
	109	L Middle temporal gyrus	−60	−9	−18	−6.02
	52	L Middle occipital gyrus	−45	−75	33	−5.64
	11	R Cerebellum	42	−45	−27	−4.15
	13	L Temporal pole	−36	15	−24	−4.04
*2*	**UNFAMILIAR OBJECTS > FAMILIAR OBJECTS AND UNFAMILIAR FACES**					
	1268	R Inferior temporal gyrus	57	−63	−12	7.04
	673	L Middle temporal gyrus	−45	−63	0	5.98
	277	R Superior occipital gyrus	27	−81	36	5.60
	140	L Superior occipital gyrus	−21	−75	42	5.47
*2*	**FAMILIAR OBJECTS AND UNFAMILIAR FACES > UNFAMILIAR OBJECTS**					
	456	L Postcentral gyrus	−51	−21	18	−6.40
	103	L Superior temporal gyrus	−51	3	−6	−5.34
	456	R Insula	36	12	−9	−4.85
	77	L Hippocampus	−33	−9	−24	−4.77
	72	R Middle temporal gyrus	54	−39	0	−4.49
	55	R Parahippocampal gyrus	30	−6	−27	−3.86
	30	R Putamen	24	12	−9	−3.65
	33	R Cuneus	9	−90	6	−3.55
	39	L Cuneus	−3	−93	15	−3.55
*3*	**UNFAMILIAR FACES > FAMILIAR FACES AND FAMILIAR OBJECTS**					
	624	R Precuneus	6	−72	42	5.45
*3*	**FAMILIAR FACES AND FAMILIAR OBJECTS > UNFAMILIAR FACES**					
	636	R Parahippocampal gyrus	30	−33	−18	−6.44
	440	L Parahippocampal gyrus	−27	−18	−21	−5.71
	170	L Cerebellum	−6	−54	0	−5.30
	141	L Temporal pole	−42	9	−30	−4.28
	57	R Inferior frontal gyrus	39	24	−15	−3.91
	93	R Middle occipital gyrus	27	−93	18	−3.68

Note: Only clusters evident during the peak timepoint (TR 3) with a bootstrap ratio of greater than +/−3.5 are reported.

* Cluster size (k) indicates the number of voxels comprising the cluster; only clusters with a minimum extent of 10 voxels are reported. BSR, Bootstrap ratio; LV, Latent Variable; L, left; R, right.

The second LV explained 33.08% of the crossblock covariance (*p* < 0.001) and appeared to primarily distinguish the *unfamiliar objects* condition from the *familiar objects* and *unfamiliar faces* conditions. The regions associated more with the *unfamiliar objects* condition relative to the *familiar objects* and *unfamiliar faces* conditions included right inferior temporal and superior/middle occipital gyrus (shown in red in Figure 2B). The regions associated with the *familiar objects* and *unfamiliar faces* conditions relative to the *unfamiliar objects* condition included the right anterior hippocampus and bilateral insular cortex (shown in blue in Figure 2B). The *familiar faces* condition was not significantly associated with either of these two patterns of activation in LV 2.

Finally, the third LV was of most interest to the current study, as it differentiated between the conditions involving familiar and unfamiliar stimuli (explaining 24.39% of the crossblock covariance, *p* < 0.048). Specifically, this LV highlighted one pattern of activation that was correlated more with the *familiar faces* and *familiar objects* conditions relative to the *unfamiliar faces* condition (shown in blue in Figure 2C), and another correlated with the *unfamiliar faces* condition relative to the two familiar conditions (shown in red in Figure 2C). The *unfamiliar objects* condition was not significantly associated with either of these two negatively-correlated activation patterns. The regions associated with the familiar conditions consisted of strong bilateral activation along the entire extent of the parahippocampal gyrus, extending into the anterior hippocampus, and also included the bilateral temporal poles. This activation corresponded well with the MTL regions identified as being sensitive to stimulus familiarity in Barense et al. (2011). In contrast, the *unfamiliar faces* condition was associated primarily with bilateral medial parietal activation.

### Seed PLS analysis

**Figure 3:**
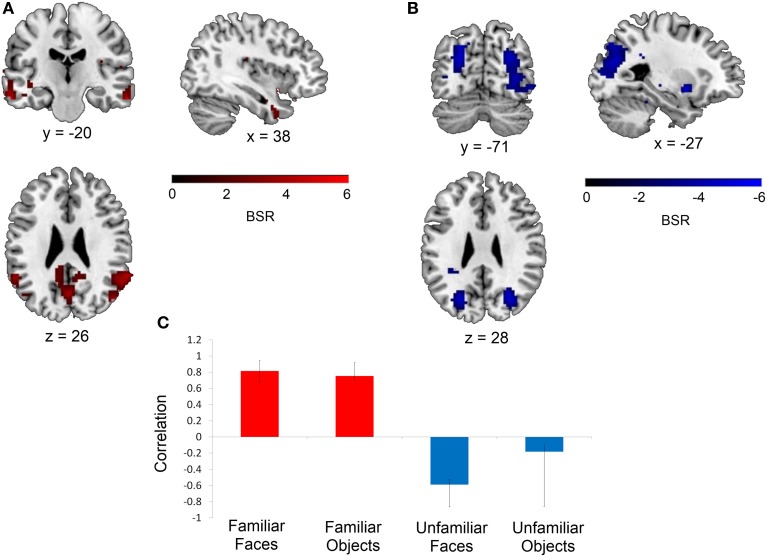
**Regions functionally connected with the right perirhinal cortex seed.** A seed PLS analysis demonstrated that the functional connectivity of the right perirhinal cortex differed depending on stimulus familiarity. **(A)** Regions shown in red are those with which the right perirhinal cortex seed was functionally connected during the two familiar conditions in TR 3, whereas **(B)** displays regions shown in blue with which the perirhinal seed was correlated during the two unfamiliar conditions in TR 3. The bar graph in **(C)** depicts the correlation of the signal in the perirhinal seed with the two networks during the four experimental conditions. Maps are thresholded at the equivalent of *p* < 0.05 for visualization purposes, and networks are overlaid on the ch2bet template in MRIcron (Rorden et al., 2007). BSR, bootstrap ratio. Error bars reflect 95% confidence intervals.

Following this mean-centered task analysis, we conducted our non-rotated seed PLS analysis with signal intensity values extracted from the mean-centered task PLS analysis for each seed. This analysis revealed that the functional connectivity of two of the eight seeds (the right perirhinal cortex and the right anterior hippocampus) was modulated by stimulus familiarity, such that these regions were functionally interacting with different networks depending on whether the stimuli to be discriminated were familiar or unfamiliar. Specifically, the right perirhinal cortex seed (*x*, *y*, *z* = 36, −9, −30) displayed this pattern (16.43% of crossblock covariance, *p* < 0.016). Figure 3 illustrates that during the two familiar conditions (shown in red), signal in the right perirhinal cortex was highly correlated with a bilateral network including lateral temporal, anterior temporal, and medial and lateral parietal cortex (see Table 3). In contrast, during the two unfamiliar conditions, the functional connectivity of this right perirhinal seed region was significantly different, and instead correlated with large sections of occipital cortex.

**Table 3:** **Regions showing significant functional connectivity with the right perirhinal cortex seed**.

Cluster size (*k*)^*^	**Brain region**	***x***	***y***	***z***	**BSR**
**REGIONS FUNCTIONALLY CONNECTED DURING THE FAMILIAR RELATIVE TO UNFAMILIAR CONDITIONS**					
442	R Superior temporal gyrus/Angular gyrus	60	−57	18	5.93
44	R Superior temporal gyrus	57	−30	12	5.24
56	R Inferior/Middle temporal gyrus	63	−18	−24	4.79
151	L Middle temporal gyrus	−42	−51	12	4.66
54	R Superior temporal gyrus	51	−6	6	4.35
70	L Inferior temporal gyrus	−60	−21	−21	4.34
241	L Cuneus	0	−69	30	4.05
86	R Temporal pole	42	3	−30	3.62
59	L Angular gyrus	−42	−69	36	3.53
37	R Precuneus	18	−48	33	3.53
**REGIONS FUNCTIONALLY CONNECTED DURING THE UNFAMILIAR RELATIVE TO FAMILIAR CONDITIONS**					
329	L Superior occipital gyrus	−18	−81	42	−5.73
740	R Middle occipital gyrus	54	−69	−9	−4.51
31	L Putamen	−27	3	−6	−4.47

Only clusters evident during the peak timepoint (TR 3) with a bootstrap ratio of greater than +/−3.5 are reported.

* Cluster size (k) indicates the number of voxels comprising the cluster; only clusters with a minimum extent of 10 voxels are reported. BSR, Bootstrap ratio; L, left; R, right.

**Figure 4:**
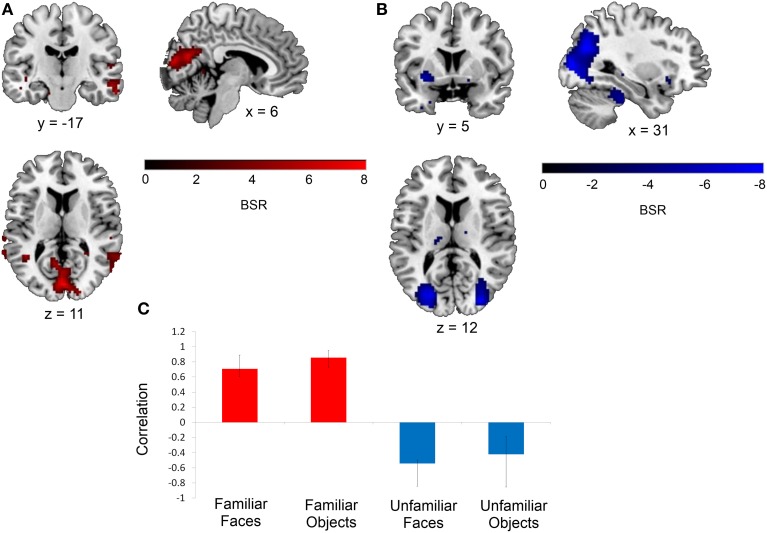
**Regions functionally connected with the right anterior hippocampal seed.** The functional connectivity of the right anterior hippocampus also differed depending on stimulus familiarity. **(A)** Regions shown in red are those with which the right anterior hippocampal seed was functionally connected during the two familiar conditions in TR 3. **(B)** Regions shown in blue are those with which the seed was correlated during the two unfamiliar conditions in TR 3. The bar graph in **(C)** depicts the correlation of the signal in the anterior hippocampal seed with the two networks during the four experimental conditions. Maps are thresholded at the equivalent of *p* < 0.05 for visualization purposes. BSR, bootstrap ratio. Error bars reflect 95% confidence intervals.

**Figure 5:**
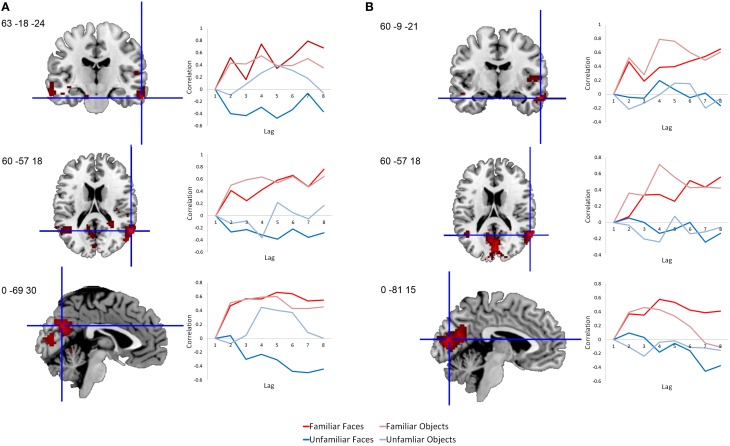
**Time course of correlations between the right perirhinal cortex and anterior hippocampus and selected functionally-connected regions.** Line graphs illustrate the correlations over time following stimulus onset between signal in the seed regions and selected regions within their functionally-connected networks. **(A)** Correlations are shown between the right perirhinal cortex seed and sample peak regions within its functionally-connected network for the two familiar conditions. **(B)** Correlations are shown between the anterior hippocampus seed and sample peak regions within its functionally-connected network for the two familiar conditions. Each lag is equivalent to a TR, which in this case is 2 s in duration. Voxel salience maps are thresholded at the equivalent of *p* < 0.05 for visualization purposes, and are overlaid on the ch2bet template in MRIcron (Rorden et al., 2007).

This same contrast of the familiar vs. unfamiliar conditions was also significant in the right anterior hippocampal seed (*x*, *y*, *z* = 27, −15, −18, 15.38% of crossblock covariance, *p* < 0.036). Like the right perirhinal cortex, the right anterior hippocampus showed differential functional connectivity for the familiar vs. unfamiliar conditions, and many of the regions identified in the two functionally-connected networks overlapped with those found in the two networks showing connectivity with the right perirhinal cortex (see Table 4 and Figure 4). During the familiar conditions, the network associated with the right anterior hippocampus included right anterior lateral temporal and lateral parietal cortex, cuneus and precuneus. For the unfamiliar conditions, the functionally-connected network consisted of regions such as bilateral occipital cortex and fusiform gyrus. Figure 5 shows the time course of the correlation between signal in the right perirhinal cortex and right anterior hippocampal seeds and a sample of the regions identified as functionally connected during the familiar conditions.

**Table 4:** **Regions showing significant functional connectivity with the right anterior hippocampal seed**.

Cluster size (*k*)^*^	**Brain region**	***x***	***y***	***z***	**BSR**
**REGIONS FUNCTIONALLY CONNECTED DURING THE FAMILIAR RELATIVE TO UNFAMILIAR CONDITIONS**					
382	L Cuneus	0	−81	15	4.94
177	R Superior temporal gyrus	60	−57	18	4.67
70	R Middle temporal gyrus	60	−9	−21	4.56
41	R Middle temporal gyrus	60	−39	−6	4.12
21	L Middle temporal gyrus	−42	−51	15	3.82
15	L Inferior temporal gyrus	−57	−24	−21	3.75
48	L Middle temporal gyrus	−66	−51	0	3.63
**REGIONS FUNCTIONALLY CONNECTED DURING THE UNFAMILIAR RELATIVE TO FAMILIAR CONDITIONS**					
1023	R Middle occipital gyrus	33	−84	6	−6.93
150	L Middle occipital gyrus	−30	−81	9	−6.14
63	L Fusiform gyrus	−48	−42	−15	−5.43
55	L Putamen	−30	6	−6	−4.66
31	L Fusiform gyrus	−36	−21	−24	−4.13
10	Parahippocampal gyrus	−24	0	−33	−4.12

Only clusters evident during the peak timepoint (TR 3) with a bootstrap ratio of greater than +/−3.5 are reported.

* Cluster size (k) indicates the number of voxels comprising the cluster; only clusters with a minimum extent of 10 voxels are reported. BSR, Bootstrap ratio; L, left; R, right.

In these same two perirhinal and anterior hippocampal regions in the left hemisphere, this contrast did not reach significance. Functional connectivity was not significantly modulated by familiarity in either the left perirhinal cortex (*x*, *y*, *z* = −33, −12, −27, 11.14% of crossblock covariance, *p* < 0.317) or the left anterior hippocampus (*x*, *y*, *z* = −21, −9, −18, 14.05% of crossblock covariance, *p* < 0.078) seeds. Similarly, the contrast of connectivity for the familiar conditions vs. the unfamiliar conditions was not significant in either hemisphere for the other two remaining seeds in the posterior hippocampus and temporal pole. Permutation testing for the LVs in the right posterior hippocampus (*x*, *y*, *z* = 33, −33, −12, 8.41% of crossblock covariance, *p* < 0.884), left posterior hippocampus (*x*, *y*, *z* = −33, −27, −15, 12.73% of crossblock covariance, *p* < 0.158), right temporal pole (*x*, *y*, *z* = 63, 3, −18, 8.94% of crossblock covariance, *p* < 0.828), and left temporal pole (*x*, *y*, *z* = −36, 18, −27, 12.92% of crossblock covariance, *p* < 0.152) indicated that the functional connectivity of these four seed regions did not differ depending on stimulus familiarity.

## Discussion

The aim of this study was to examine the functional connectivity of several MTL structures during a complex perceptual discrimination task. Specifically, we were interested in whether MTL functional connectivity during the task would be affected by participants' prior familiarity with the stimuli to be discriminated. The perirhinal cortex, anterior and posterior hippocampus, and temporal pole were previously shown to be more active during perceptual discrimination of familiar, relative to unfamiliar, faces and objects (Barense et al., 2011). We anticipated that when participants discriminated between familiar stimuli, some or all of these areas would show increased connectivity with anterior temporal, lateral temporal, and inferior parietal regions known to be involved in semantic memory (Binder and Desai, 2011), relative to when participants discriminated between novel or unfamiliar stimuli. Our findings indicate that the functional connectivity of the right perirhinal cortex and right anterior hippocampus did in fact differ across familiar and unfamiliar conditions, while the connectivity of the left perirhinal cortex, left anterior hippocampus, and bilateral posterior hippocampus and temporal pole was unaffected by stimulus familiarity.

During the two familiar conditions, signal in the right perirhinal cortex covaried with signal in bilateral anterior portions of the middle and inferior temporal gyri, the right temporal pole, bilateral posterior aspects of the middle and superior temporal gyri, and bilateral angular gyrus, cuneus and precuneus. In contrast, during the unfamiliar conditions, the right perirhinal cortex instead showed connectivity with bilateral occipital cortex (Figure 3). The pattern of differential functional connectivity exhibited by the right anterior hippocampus was nearly identical to that of the right perirhinal cortex for both the familiar and unfamiliar conditions, though during the familiar conditions, the network associated with the right anterior hippocampus was slightly more right-lateralized (Figure 4).

## Relationship of observed functional connectivity to anatomical connectivity

The regions identified as being functionally connected to the perirhinal cortex and anterior hippocampus during perceptual discrimination correspond well with what is already known about the anatomical connections of the MTL. Functional correlations between regions during resting states are thought to reflect intrinsic anatomical connections, and studies using such methods have demonstrated that the perirhinal cortex and anterior hippocampus have very similar intrinsic functional connectivity, while the parahippocampal gyrus and posterior hippocampus show functional correlations with a separate, more posterior network (Kahn et al., 2008; Ranganath and Ritchey, 2012). Given the similar resting-state connectivity of the perirhinal cortex and anterior hippocampus, it therefore is not surprising that the task-related functional connectivity of these two regions was similarly affected by stimulus familiarity. The perirhinal cortex and anterior hippocampus are anatomically associated with an anterior cortical pathway that encompasses anterior lateral temporal cortex, including the temporal poles and following along the middle temporal gyrus (Kahn et al., 2008). Moreover, the perirhinal cortex has intrinsic connectivity with anterior fusiform gyrus, anterior lateral and inferior temporal cortex, anterior hippocampus, temporoparietal cortex, and multiple aspects of prefrontal cortex (Libby et al., 2012).

## Regions functionally connected to the perirhinal cortex and hippocampus

The networks that correlated with the perirhinal cortex and anterior hippocampus during the familiar face and familiar object conditions included anterior temporal, lateral temporal, and inferior parietal regions, which are all areas thought to represent semantic information. In particular, parts of the inferior parietal lobe and significant portions of the ventral and lateral temporal lobes are thought to be high-level “convergence zones” in which represented information is abstract and independent of specific modalities (Binder and Desai, 2011; Fairhall and Caramazza, 2013). The anterior temporal lobes have also been proposed to serve as an amodal semantic hub (Binney et al., 2010; Visser et al., 2010), representing conceptual similarities among items that differ drastically in shape, color, and function (e.g., the similarities between an ostrich and a hummingbird; Rogers et al., 2004; Patterson et al., 2006, 2007). Additionally, it has been suggested that the anterior temporal lobes store representations of unique entities, as anterior temporal lobe activation is often seen in response to the recognition of specific familiar or famous faces (e.g., Gorno-Tempini et al., 1998; Leveroni et al., 2000; Damasio et al., 2004) and famous buildings (Gorno-Tempini and Price, 2001), though others have argued that a more accurate interpretation of anterior temporal lobe function is in the representation of abstract social knowledge (Olson et al., 2007, 2013).

Within these broad regions, the specific structures identified in the functionally-connected networks have already been associated with semantic tasks involving face and object stimuli. For example, retrieving non-lexical information about the professions associated with famous faces produced activation in anterior middle temporal gyrus, temporoparietal junction, and temporal pole, while successful naming of famous faces generated activation in left posterior middle temporal gyrus and left inferior parietal cortex (Gesierich et al., 2012). All of these regions were present in the functionally-connected networks associated with the familiar conditions in the present study. Also identified in these networks was the angular gyrus, which has been described as a heteromodal region that is capable of integrating a wide range of conceptual information, and is consistently activated by a variety of semantic concepts with different modality-specific associations (Bonner et al., 2013).

The connectivity of the perirhinal cortex and anterior hippocampus with the cuneus for the familiar conditions may have been driven primarily by participants automatically activating conceptual knowledge about the familiar objects, as cuneus activation is seen in semantic tasks requiring participants to retrieve knowledge about the proper use of objects (Ebisch et al., 2007) and when judging the semantic relatedness of words referring to tools (Tyler et al., 2003). The connectivity with the precuneus, which is a region most frequently associated with the act of episodic memory retrieval accompanied by rich visual imagery (Cavanna and Trimble, 2006), could reflect spontaneous retrieval of episodic material associated with the familiar stimuli. Similarly, the presence of the calcarine fissure in the familiar face and object networks may have resulted from a greater degree of mental imagery generated for items with which participants are familiar (Klein et al., 2000; Lambert et al., 2002).

## Hemispheric differences

As the intrinsic functional connectivity of the perirhinal cortex and anterior hippocampus is similar in both hemispheres (Libby et al., 2012), and both left and right perirhinal cortex and anterior hippocampus were significantly more active for familiar vs. unfamiliar discriminations (Barense et al., 2011), it is not entirely clear why the effect of stimulus familiarity only significantly impacted the functional connectivity for these regions in the right hemisphere. As mentioned previously, the effect did approach significance in the left anterior hippocampus. However, it is possible that the automatic retrieval of semantic information associated with non-verbal stimuli in the current perceptual discrimination paradigm is slightly more lateralized to the right hemisphere. Previous studies have shown that the recognition of familiar faces and subsequent retrieval of person-related conceptual knowledge has a tendency to show rightward lateralization, being particularly associated with activation in the right anterior temporal lobes (Gainotti, 2013).

## Category selectivity

The functional connectivity of each of the eight seed regions was not impacted in the same way by stimulus familiarity. The reason for this differential functional connectivity likely stems from some degree of stimulus category selectivity within these seed regions. Although all seeds were shown in Barense et al. (2011) to be sensitive in some manner to stimulus familiarity during perceptual discrimination, this varied for each seed depending on the stimulus category (i.e., faces vs. objects). More specifically, while the bilateral perirhinal cortex and anterior hippocampus were more active for familiar vs. unfamiliar stimuli in general (irrespective of whether the stimuli were faces or objects), the temporal pole was only sensitive to the familiarity of faces, and the posterior hippocampus was sensitive only to the familiarity of objects. Therefore, it seems likely that while all of these regions are involved to some extent in representing semantic information about the stimuli to be discriminated, this involvement is modulated by stimulus category and therefore the functional connectivity of these regions will not necessarily be identical.

## Mean-centered task PLS LV1 and LV2: effects of stimulus category

The mean-centered task PLS analysis, conducted as a preliminary step from which to extract signal intensity values for the functional connectivity analyses, also highlighted the main factors explaining the covariance between the functional neuroimaging data and the experimental design. This analysis identified regions associated with optimal combinations of the *familiar faces*, *unfamiliar faces*, *familiar objects*, and *unfamiliar objects* conditions. The first two latent variables primarily distinguished between specific stimulus categories irrespective of their familiarity. LV1 highlighted differences between the face and object conditions, and the existence of such differences is unsurprising given the numerous dissimilarities between faces and objects. LV2 distinguished the *unfamiliar objects* (greebles) condition from the remaining three conditions involving more everyday items, suggesting that there is a fundamental difference between discriminations involving a completely novel type of stimulus that has never been encountered before compared to discriminations involving stimuli to which participants have had some exposure—whether it be to the stimulus category in general (e.g., faces in general) or to the actual exemplars themselves (e.g., specific famous faces).

In contrast to these first two LVs, LV3 revealed differences in neural representation that resulted from the varying levels of stimulus familiarity, irrespective of stimulus type, which was our main *a priori* factor of interest. The relative importance of these three LVs indicate that while differences among stimulus categories account for a greater degree of covariance than differences in stimulus familiarity, both of these constructs make significant and simultaneous contributions.

## Mean-centered task PLS LV3: effects of stimulus familiarity

The focus of the current study was on the familiarity-related effects identified in the third LV. Nevertheless, some of the regions identified in the first latent variable were also relevant to this issue. The first LV identified the anterior hippocampus and anterior lateral temporal cortex as being particularly involved in making perceptual discriminations among familiar faces, relative to discriminations among familiar objects. The fact that this activation was located more anteriorly in the temporal lobes supports the findings of previous studies demonstrating some degree of stimulus selectivity along the longitudinal axis of the MTL, such that anterior regions are more content-general, responding to face, object, and some scene stimuli, while posterior regions respond selectively to scenes (Litman et al., 2009; Liang et al., 2012). Moreover, the anterior hippocampal and anterior lateral temporal activation seen in response to familiar faces corroborates the findings of Trinkler et al. (2009), who found that activation in the anterior hippocampus and anterior middle temporal gyrus was associated with greater pre-experimental knowledge about face stimuli, and that anterior hippocampal activation increased with the degree to which the faces were personally-known to participants. The anterior hippocampal activation seen for the *familiar faces* condition in the present study may therefore reflect the retrieval of episodic autobiographical information associated with the famous individuals whose faces were presented. While the current implication of the anterior hippocampus in perceptual discrimination of familiar faces is consistent with previous findings (e.g., Lee et al., 2008), it is at odds with a recent suggestion that a bias toward pattern completion processes in the anterior hippocampus renders it of no use for fine perceptual discrimination tasks (Poppenk et al., 2013). The perirhinal cortex alone does in fact appear to be capable of making such discriminations among object and face stimuli (Barense et al., 2010a), and the involvement of the anterior hippocampus may therefore simply stem from its strong connectivity with the perirhinal cortex (Kahn et al., 2008; Libby et al., 2012). However, the repeated implication of the anterior hippocampus in the perceptual discrimination of faces suggests the possibility of an additional, as yet unspecified, role for the anterior hippocampus in such tasks.

The third latent variable implicated large sections of the MTL in perceptual discriminations of familiar stimuli in general. For the combination of the *familiar faces* and *familiar objects* conditions, activation was localized bilaterally along the entire extent of the parahippocampal gyrus, extending into the anterior hippocampus, perirhinal cortex, and slightly into the posterior hippocampus, as well as in the temporal poles. These regions are generally consistent with those identified as showing greater activity during the familiar vs. unfamiliar conditions in the univariate general linear model (GLM) used by Barense et al. (2011), suggesting that the multivariate PLS and univariate GLM methods detected similar effects. Widespread involvement of the MTL in perceptual discrimination offers support for the proposition that aspects of this region serve as an extension of a representational hierarchy in the ventral visual stream (Cowell et al., 2010; Lee et al., 2012). Moreover, the observed sensitivity of the MTL to stimulus familiarity in perceptual discrimination suggests that these regions also represent semantic information (Murray and Bussey, 1999; Taylor et al., 2006; Barense et al., 2011).

## Implications for semantic dementia and MTL amnesia

The current findings offer insight into an intriguing and previously unexplained pattern of results from Barense et al. (2010b). As mentioned previously, this study found that two patient groups—amnesics with non-progressive MTL damage (“MTL amnesics”) and patients with semantic dementia (“SD patients”)—were both impaired relative to controls at perceptual discriminations of complex and visually similar stimuli. However, only the controls and MTL amnesics showed a benefit from stimulus familiarity when making such discriminations. By contrast, the SD patients failed to show this facilitation from the use of familiar stimuli, likely because they were unable to engage support from their impaired semantic system. Nonetheless, the neuroanatomical correlates of these behavioral differences were unclear. The two patient groups had largely overlapping MTL damage, particularly in the perirhinal cortex. Additionally, although Barense et al. (2011) illustrated that multiple MTL subregions were sensitive to stimulus familiarity during perceptual discrimination, none of these regions were clearly more damaged in the SD patients than the MTL amnesics. The current results suggest that even though the ability to discriminate between items with overlapping features depends heavily upon the perirhinal cortex, this structure (and those structures with which it is closely connected, such as the anterior hippocampus) receives relevant input from other functionally-connected regions depending on the familiarity of the stimuli to be discriminated. Both the SD patients and the MTL amnesics had damage to the perirhinal cortex, which impaired their discrimination performance, but the SD patients' additional damage to anterior and lateral temporal cortex, identified here as being functionally connected with the perirhinal cortex and anterior hippocampus for familiar stimuli, may have prevented them from benefitting from the support provided by intact access to semantic memory.

## Conclusions

In summary, during a perceptual discrimination task in which participants selected the odd-one-out from a set of three complex faces or objects with many shared visual features, the functional connectivity of the right perirhinal cortex and right anterior hippocampus was significantly modulated by the degree to which participants were familiar with the stimuli being discriminated. This was the case despite the fact that the task did not explicitly require the retrieval of any conceptual information about the items to be discriminated and could be completed without drawing upon semantic memory. For familiar relative to unfamiliar faces and objects, both the right perirhinal cortex and right anterior hippocampus showed enhanced functional correlations with a network of regions associated with semantic knowledge. These findings illustrate that experience and expertise with particular classes of objects influences not only the location of their neural representation (McKeeff et al., 2010; McGugin et al., 2012), but also the functional interactions of these representations with broader whole-brain networks. The results have potential implications for semantic dementia patients, as the results suggest that it was the patients' inability to engage a network of regions including the lateral temporal cortex and the temporal pole, as opposed to localized damage in the perirhinal cortex or hippocampus, that impaired their ability to benefit from the use of familiar stimuli in non-semantic perceptual tasks (Binder and Desai, 2011). Future research in semantic dementia showing diminished interactions between perirhinal cortex, anterior hippocampus, and anterior/lateral temporal regions relative to normal controls during perceptual discrimination tasks will confirm this hypothesis.

## Author contributions

Morgan D. Barense was responsible for the original experimental design, programming, and fMRI data collection. The data analysis was completed by Victoria C. McLelland with assistance from David Chan and Morgan D. Barense. Victoria C. McLelland interpreted the results and wrote the paper with input from Morgan D. Barense, Susanne Ferber, and David Chan.

### Conflict of interest statement

The authors declare that the research was conducted in the absence of any commercial or financial relationships that could be construed as a potential conflict of interest.
